# Supplementary material for: Inter-strain diversity among coastal picocyanobacteria across salinity gradients
Source: PLoS One. 2026 Oct 7;21(10):e0335835. doi: 10.1371/journal.pone.0335835 (PMC13645077; doi:10.1371/journal.pone.0335835)
Supplement: Supplementary file 1 [file pone.0335835.s017.docx]

Reviewer #1: Review of D-25-56126

*Dear Reviewer,*

*thank you very much for the helpful comments. We changed and responded to them accordingly.*

General comments

The present manuscript presents the results from a Factorial (almost Response Surface) experimental design to study optimal ranges of salinity and light for several strains of picocyanobacterial. The main parameters analyzed as a response to salinity x light were biovolume, chlorophyll a, and fluorescence. The strains were initially selected in order to represent a salinity gradient, based their habitats of origin. In addition, it was performed a genomic study, which main objective was to find patterns of adaptation to the salinity gradient as well.
The results show a clear pattern of adaptation in terms of the different measures of growth optima. But there are no main patterns in terms of genomic adaptation.
I would recommend the publication of the present manuscript after the correction of several minor aspects detailed below.

Specific comments

- Line 49: I guess it should be ‘clades’ not ‘claded’.

*Changed accordingly, see line 47.*

- Lines 91-92: This statement, establishing the main hypothesis of this study has to be corrected, since the authors did not perform a strict test for the presence of the meta-populations they referred to. Their findings are restricted to a single cell isolated from each of the habitats. Of course, if the adaptations to salinity found in the strains match the salinity gradient of their habitat of origin (as it was the case for the physiology but not the genomics), that should be explained, most likely, by the presence of adapted meta-populations. But, strictly, the meta-populations were not studied, only one clone from each habitat.
In my opinion, this reference to the meta-populations should be made in the discussion, but not set in the hypothesis.

*Changed accordingly, see line 98.*

- Lines 111-112: I don’t understand the reason for growing those two strains in freshwater (when they are originally brackish) and then shifting to 4 ppm salinity.

*We changed these sentences accordingly, see line 116. Those two strains were maintained in freshwater at the original culture collection, but we moved them up to higher salinity, as they were growing better in slightly brackish medium.*

- Line 118: if you used Milli-Q water, you can skip the term “deionized water”.

*We would prefer to keep this wording and also not use Milli-Q (as it is a brand name).*

- Line 130: the authors make a reference here light levels, for the first time. This confusing for the reader, because the light treatment was not previously described, as it was the salinity. It is described in the next sentence. This sentence should go earlier.

*Changed accordingly, see line 139*.

- Line 166: The Gompertz equation does not show in my pdf. Similar problems in lines 186-187, 211-218 and other places throughout the manuscript.

*We re-exported it and attached a pdf with a working equation.*

- Line 369: the “l” of the Cl ion, is written as a superindex.
*Changed accordingly, see line 403.*
- Lines 434-451: There is very little, if any, discussion in this paragraph, it is mainly a repetition of things that have been said, differently, in the Results. If it contains new aspects here, they should be anyways moved to the Results.

*Thanks for highlighting this issue. We moved parts of the discussion to the results and expanded the discussion with more cited literature. Please refer to the track-changed version to see the differences.*

*Dear Reviewer,*

*Thank you very much for those thoughtful and extensive comments. We re-wrote large parts of our results and discussion section and also referred to suggested literature where useful. We respond to specific comments with line numbers. More extensive changes are highlighted in our track-change version.*

Reviewer #2: The study by Berthold et al. is a physiological study of salinity and PAR response of five picocyanobacterial strains. The study also investigated the genetic capacity of osmoregulation in the strains. The study is a valuable contribution to the increasing interest in brackish microorganisms diversity and adaptation. I encourage the authors to develop the presentation of their data and the discussion especially in the context of the brackish environment and physiological response of the diverse isolates.

In the abstract the authors mention acclimation to different conditions and that Chla can vary. In this context, biovolume may also vary across different conditions. Cell size and temperature relationships are well established in literature, and similarly light and chla, but the physiological response across salinity gradients is less explored to which this study brings new important knowledge. In the current version of the manuscript, the GAM approach, although highly relevant, prevents a discussion on the physiological adaptation in terms of Chl a content, and cell size which I suggest to expand. For example, was there a consistent trend in the adaptation across the different isolates or did it vary depending on metabolic strategies?

The introduction and discussion could be improved by including more information about the brackish context in relation to diversity and adaptation to gradients in light, temperature, nutrients and salinity. On the macroorganism level, brackish conditions are associated with smaller organism size (e.g. mussels) and less diversity. However, on the microorganism level, brackish environments harbor a unique and high diversity of organisms. See for example Mazur-Marzec et al. 2024 FEMS Microbiology Reviews, Volume 48, <https://doi.org/10.1093/femsre/fuae024>; Herlemann et al. 2011 The ISME Journal 5, 571–1579 (2011) <https://www.nature.com/articles/ismej201141>. Similarly, the brackish picocyanobacterial composition seems to be much more diverse compared to other environments (>2000 16S ASVs Alegria-Zufia et al. 2022 Sci Rep 12:14330. 10.1038/s41598-022-18454-8). Further, in the context of genomic strategies of salinity tolerance the authors may want to refer to the study by Cabello Yeves et al. BMC Biology (2022) 20:175 <https://doi.org/10.1186/s12915-022-01379-z>

Large parts of the discussion section would be a better fit for the results section. The results section largely describes the patterns observed in the figures but the overall patterns in the results are more clearly presented in the discussion, including a comparison between the strains which I found well written. A more thorough contextualization of the new knowledge presented in this study compared with previous studies and larger implications would also strengthen the discussion. In the abstract, large emphasis is put on metabolic capacity and rate, but this is not well supported in the results and discussion.

Furthermore, the section on optical attenuation in the discussion is not well connected with the findings of the study or the knowledge of strain specific light niches and response in pigmentation to salinity and light (see e.g. Aguilera et al. 2023 Environ Microbiol 25: 1674-1695 <https://doi.org/10.1111/1462-2920.16384)>. Here the additional knowledge of the different physiological response among the strains investigated in the study could be more clearly presented.

I would find it helpful with a table summarizing the characteristics of each of the tested strains, for example, place of isolation, clade, pigmentation, genome size, original reference, accession number.

There was a formatting issue with formulas and symbols being replaced by squares in the version that I have read.

Specific comments:

Line 43-44: This sentence is written as if a small genome would preclude within clade diversity. Please rephrase.

*Changed accordingly, see line 43.*

Line 47-49: The clade division gets complicated. Rather than mentioning Synechococcus and Cyanobium as a clade I would suggest using the established cluster 5 (5.1, 5.2 and 5.3).

*Thanks for the suggestion. We added the information, where available.*

Line 70: Here you mention salinity but in the context of the paragraph other parameters such as temperature and light may be more relevant.

*Changed accordingly, see line 70.*

Line 81-83: This sentence is unclear. It sounds like there is a problem that there are more isolates and data for these.

*Re-phrased the sentence, see line 82.*

Line 88-89: All prokaryotes are “related based on conserved sequences including 16s rDNA”. Please be more specific to address why these particular strains were included in the study.

*The choice of strains is now addressed:*

*"..originating from geographically and ecologically diverse regions of the Gulf of Mexico, Baltic Sea, Chesapeake Bay, and coastal Pacific. We chose these particular strains as they are either isolated from similar ecosystems, or are already included in physiological or genomic studies elsewhere, allowing us to validate our findings." see line 91 ff.*

Line 91: “across brackish environments” unclear, are you referring to the brackish microbiome or salinity gradients from marine, brackish to freshwater.

*Changed accordingly, see line 93.*

Line 365: “in study picocyanobacterial” revise to “for the picocyanobacterial in this study”

*Changed as suggested., see line 386*

Line 372: “different saline environments” unclear.

*Changed accordingly., see line 366.*

Line 374: Please add a reference.

*Added a reference.*

Line 383-385: Please see Cabello Yeves et al. BMC Biology (2022) 20:175 <https://doi.org/10.1186/s12915-022-01379-z>

*Thanks, we added that one, too.*

Line 419-420: This statement implies that this is the only mechanism. Please rephrase.

*Changed while re-writing the section.*

**Other Changes:**

For clarity abbreviations for metabolites glycosyl glycerol, glycosyl glycerate, and glycine betaine were replaced with the full terms.
