# Supplementary material for: Inter-strain diversity among coastal picocyanobacteria across salinity gradients
Source: PLoS One. 2026 Oct 7;21(10):e0335835. doi: 10.1371/journal.pone.0335835 (PMC13645077; doi:10.1371/journal.pone.0335835)
Supplement: Supplementary file 2 [file pone.0335835.s018.docx]

Reviewer #1: Review of D-25-56126

*Dear Reviewer,*

*Thank you for your eview.*

Reviewer #2
*Dear Reviewer,*

*Thank you for your constructive comments and suggestions to improve our manuscript. We apologize for failing to explicitly comment on all general comments during round 1. Thus, we re-evaluated some of the points raised, and added some more parts to the discussion.*

Reviewer #2: Thank you for the revision. I have previously reviewed this manuscript and I believe that it is a valuable contribution with solid experimental data addressing coupling between physiological adaptation and genomics of brackish picocyanobacteria.

Thank you.

Although the authors have responded to my minor specific comments, they have not clearly addressed my general comments in the response letter or in the revised manuscript. This includes a suggestion of expanding the discussion on the physiological adaptation in terms of Chl a content, and cell size as well as a more thorough description about the brackish context in relation to diversity and adaptation to gradients in light, temperature, nutrients and salinity. I understand that not all suggestions have to be fully integrated in a revised manuscript but I would appreciate a clear response from the authors.

We expanded the discussion now to include cell size influences potential colony formation. However, we did not expand the discussion on Chl a content, temperature or nutrients besides what is already present in the manuscript. We see them as important, but they were not the major focus of this study. After this salinity-focussed data set we accumulated similar datasets on temperature, nutrients and photoperiods for manuscript(s) in the future, but would be too much for just one manuscript.

From your first review: In the current version of the manuscript, the GAM approach, although highly relevant, prevents a discussion on the physiological adaptation in terms of Chl a content, and cell size which I suggest to expand. For example, was there a consistent trend in the adaptation across the different isolates or did it vary depending on metabolic strategies?

We added individual GAMs for each strain to the supplement, to make strain-specific trends easier to see. Also, we highlight, that Supplement Table 2 includes strain specific biovolume estimates for each light level.

I also encourage the authors to include a conclusion about their findings and the larger implications at the end of the discussion. The last paragraph of the discussion is now on the optical attenuation, which was a section that I encouraged the authors to expand on, but it may not be the final message that the authors want to convey (does not align with the abstract or aims in the introduction).

Thank you again for the opportunity. We agree and have added a small Conclusions section to the manuscript.

There is still a problem with formulas and special signs as they show as boxes in my version (which was also pointed out by Reviewer#1).

We checked again with our own generated .pdfs, where the formulas are visible. The issue arises when the PLoS Manuscript submission system auto-generates a .pdf file from our .docx. We have uploaded the manuscript as pdf with this submission and will talk with the editorial board about the issue if the manuscript goes into production.
