# Supplementary material for: Inter-strain diversity among coastal picocyanobacteria across salinity gradients
Source: PLoS One. 2026 Oct 7;21(10):e0335835. doi: 10.1371/journal.pone.0335835 (PMC13645077; doi:10.1371/journal.pone.0335835)
Supplement: Peer review history

# Peer review: Decision Letter 0

**DOI:** 10.1371/journal.pone.0335835.r001

**Authors:** Balaji Prasath Barathan

5 Apr 2026

Dear Dr. Campbell,

Thank you for submitting your manuscript to PLOS ONE. After careful consideration, we feel that it has merit but does not fully meet PLOS ONE’s publication criteria as it currently stands. Therefore, we invite you to submit a revised version of the manuscript that addresses the points raised during the review process.

Please submit your revised manuscript by May 20 2026 11:59PM. If you will need more time than this to complete your revisions, please reply to this message or contact the journal office at plosone@plos.org. When you're ready to submit your revision, log on to https://www.editorialmanager.com/pone/ and select the 'Submissions Needing Revision' folder to locate your manuscript file.

- A letter that responds to each point raised by the academic editor and reviewer(s). You should upload this letter as a separate file labeled 'Response to Reviewers'.
- A marked-up copy of your manuscript that highlights changes made to the original version. You should upload this as a separate file labeled 'Revised Manuscript with Track Changes'.
- An unmarked version of your revised paper without tracked changes. You should upload this as a separate file labeled 'Manuscript'.

If you would like to make changes to your financial disclosure, please include your updated statement in your cover letter. Guidelines for resubmitting your figure files are available below the reviewer comments at the end of this letter.

If applicable, we recommend that you deposit your laboratory protocols in protocols.io to enhance the reproducibility of your results. Protocols.io assigns your protocol its own identifier (DOI) so that it can be cited independently in the future. For instructions see: https://journals.plos.org/plosone/s/submission-guidelines#loc-laboratory-protocols. Additionally, PLOS ONE offers an option for publishing peer-reviewed Lab Protocol articles, which describe protocols hosted on protocols.io. Read more information on sharing protocols at https://plos.org/protocols?utm_medium=editorial-email&utm_source=authorletters&utm_campaign=protocols.

As the corresponding author, your ORCID iD is verified in the submission system and will appear in the published article. PLOS supports the use of ORCID, and we encourage all coauthors to register for an ORCID iD and use it as well. Please encourage your coauthors to verify their ORCID iD within the submission system before final acceptance, as unverified ORCID iDs will not appear in the published article. *Only*  the individual author can complete the verification step; PLOS staff *cannot*  verify ORCID iDs on behalf of authors.

We look forward to receiving your revised manuscript.

Kind regards,

Barathan Balaji Prasath

Academic Editor

PLOS One

**Journal Requirements:**

When submitting your revision, we need you to address these additional requirements.

1. Please ensure that your manuscript meets PLOS ONE's style requirements, including those for file naming. The PLOS ONE style templates can be found at

https://journals.plos.org/plosone/s/file?id=wjVg/PLOSOne_formatting_sample_main_body.pdf and

https://journals.plos.org/plosone/s/file?id=ba62/PLOSOne_formatting_sample_title_authors_affiliations.pdf

2. In your Methods section, please provide additional information regarding the permits you obtained for the work. Please ensure you have included the full name of the authority that approved the field site access and, if no permits were required, a brief statement explaining why.

3. Thank you for stating the following financial disclosure:

“Natural Sciences and Engineering Research Council of Canada

Canada Foundation for Innovation

New Brunswick Foundation for Innovation

Deutsche Forschungsgemeinschaft.”

Please state what role the funders took in the study.  If the funders had no role, please state: "The funders had no role in study design, data collection and analysis, decision to publish, or preparation of the manuscript."

If this statement is not correct you must amend it as needed.

Please include this amended Role of Funder statement in your cover letter; we will change the online submission form on your behalf.

4. Please note that your Data Availability Statement is currently missing the repository name and or the DOI/accession number of each dataset OR a direct link to access each database. If your manuscript is accepted for publication, you will be asked to provide these details on a very short timeline. We therefore suggest that you provide this information now, though we will not hold up the peer review process if you are unable.

5. If the reviewer comments include a recommendation to cite specific previously published works, please review and evaluate these publications to determine whether they are relevant and should be cited. There is no requirement to cite these works unless the editor has indicated otherwise.

Additional Editor Comments (if provided):

[Note: HTML markup is below. Please do not edit.]

Reviewers' comments:

Reviewer's Responses to Questions

**Comments to the Author**

1. Is the manuscript technically sound, and do the data support the conclusions?

Reviewer #1: Yes

Reviewer #2: Partly

**********

2. Has the statistical analysis been performed appropriately and rigorously?

Reviewer #1: Yes

Reviewer #2: Yes

**********

3. Have the authors made all data underlying the findings in their manuscript fully available?

The PLOS Data policy

Reviewer #1: Yes

Reviewer #2: Yes

**********

4. Is the manuscript presented in an intelligible fashion and written in standard English?

Reviewer #1: Yes

Reviewer #2: Yes

**********

Reviewer #1: Review of D-25-56126

General comments

The present manuscript presents the results from a Factorial (almost Response Surface) experimental design to study optimal ranges of salinity and light for several strains of picocyanobacterial. The main parameters analyzed as a response to salinity x light were biovolume, chlorophyll a, and fluorescence. The strains were initially selected in order to represent a salinity gradient, based their habitats of origin. In addition, it was performed a genomic study, which main objective was to find patterns of adaptation to the salinity gradient as well.

The results show a clear pattern of adaptation in terms of the different measures of growth optima. But there are no main patterns in terms of genomic adaptation.

I would recommend the publication of the present manuscript after the correction of several minor aspects detailed below.

Specific comments

- Line 49: I guess it should be ‘clades’ not ‘claded’.

- Lines 91-92: This statement, establishing the main hypothesis of this study has to be corrected, since the authors did not perform a strict test for the presence of the meta-populations they referred to. Their findings are restricted to a single cell isolated from each of the habitats. Of course, if the adaptations to salinity found in the strains match the salinity gradient of their habitat of origin (as it was the case for the physiology but not the genomics), that should be explained, most likely, by the presence of adapted meta-populations. But, strictly, the meta-populations were not studied, only one clone from each habitat.

In my opinion, this reference to the meta-populations should be made in the discussion, but not set in the hypothesis.

- Lines 111-112: I don’t understand the reason for growing those two strains in freshwater (when they are originally brackish) and then shifting to 4 ppm salinity.

- Line 118: if you used Milli-Q water, you can skip the term “deionized water”.

- Line 130: the authors make a reference here light levels, for the first time. This confusing for the reader, because the light treatment was not previously described, as it was the salinity. It is described in the next sentence. This sentence should go earlier.

- Line 166: The Gompertz equation does not show in my pdf. Similar problems in lines 186-187, 211-218 and other places throughout the manuscript.

- Line 369: the “l” of the Cl ion, is written as a superindex.

- Lines 434-451: There is very little, if any, discussion in this paragraph, it is mainly a repetition of things that have been said, differently, in the Results. If it contains new aspects here, they should be anyways moved to the Results.

Reviewer #2: The study by Berthold et al. is a physiological study of salinity and PAR response of five picocyanobacterial strains. The study also investigated the genetic capacity of osmoregulation in the strains. The study is a valuable contribution to the increasing interest in brackish microorganisms diversity and adaptation. I encourage the authors to develop the presentation of their data and the discussion especially in the context of the brackish environment and physiological response of the diverse isolates.

In the abstract the authors mention acclimation to different conditions and that Chla can vary. In this context, biovolume may also vary across different conditions. Cell size and temperature relationships are well established in literature, and similarly light and chla, but the physiological response across salinity gradients is less explored to which this study brings new important knowledge. In the current version of the manuscript, the GAM approach, although highly relevant, prevents a discussion on the physiological adaptation in terms of Chl a content, and cell size which I suggest to expand. For example, was there a consistent trend in the adaptation across the different isolates or did it vary depending on metabolic strategies?

The introduction and discussion could be improved by including more information about the brackish context in relation to diversity and adaptation to gradients in light, temperature, nutrients and salinity. On the macroorganism level, brackish conditions are associated with smaller organism size (e.g. mussels) and less diversity. However, on the microorganism level, brackish environments harbor a unique and high diversity of organisms. See for example Mazur-Marzec et al. 2024 FEMS Microbiology Reviews, Volume 48, https://doi.org/10.1093/femsre/fuae024; Herlemann et al. 2011 The ISME Journal 5, 571–1579 (2011) https://www.nature.com/articles/ismej201141. Similarly, the brackish picocyanobacterial composition seems to be much more diverse compared to other environments (>2000 16S ASVs Alegria-Zufia et al. 2022 Sci Rep 12:14330. 10.1038/s41598-022-18454-8). Further, in the context of genomic strategies of salinity tolerance the authors may want to refer to the study by Cabello Yeves et al. BMC Biology (2022) 20:175 https://doi.org/10.1186/s12915-022-01379-z

Large parts of the discussion section would be a better fit for the results section. The results section largely describes the patterns observed in the figures but the overall patterns in the results are more clearly presented in the discussion, including a comparison between the strains which I found well written. A more thorough contextualization of the new knowledge presented in this study compared with previous studies and larger implications would also strengthen the discussion. In the abstract, large emphasis is put on metabolic capacity and rate, but this is not well supported in the results and discussion.

Furthermore, the section on optical attenuation in the discussion is not well connected with the findings of the study or the knowledge of strain specific light niches and response in pigmentation to salinity and light (see e.g. Aguilera et al. 2023 Environ Microbiol 25: 1674-1695 https://doi.org/10.1111/1462-2920.16384). Here the additional knowledge of the different physiological response among the strains investigated in the study could be more clearly presented.

I would find it helpful with a table summarizing the characteristics of each of the tested strains, for example, place of isolation, clade, pigmentation, genome size, original reference, accession number.

There was a formatting issue with formulas and symbols being replaced by squares in the version that I have read.

Specific comments:

Line 43-44: This sentence is written as if a small genome would preclude within clade diversity. Please rephrase.

Line 47-49: The clade division gets complicated. Rather than mentioning Synechococcus and Cyanobium as a clade I would suggest using the established cluster 5 (5.1, 5.2 and 5.3).

Line 70: Here you mention salinity but in the context of the paragraph other parameters such as temperature and light may be more relevant.

Line 81-83: This sentence is unclear. It sounds like there is a problem that there are more isolates and data for these.

Line 88-89: All prokaryotes are “related based on conserved sequences including 16s rDNA”. Please be more specific to address why these particular strains were included in the study.

Line 91: “across brackish environments” unclear, are you referring to the brackish microbiome or salinity gradients from marine, brackish to freshwater.

Line 365: “in study picocyanobacterial” revise to “for the picocyanobacterial in this study”

Line 372: “different saline environments” unclear.

Line 374: Please add a reference.

Line 383-385: Please see Cabello Yeves et al. BMC Biology (2022) 20:175 https://doi.org/10.1186/s12915-022-01379-z

Line 419-420: This statement implies that this is the only mechanism. Please rephrase.

**********

what does this mean?). If published, this will include your full peer review and any attached files.

If you choose “no”, your identity will remain anonymous but your review may still be made public.

**Do you want your identity to be public for this peer review?**  For information about this choice, including consent withdrawal, please see our Privacy Policy

Reviewer #1: No

Reviewer #2: No

**********

[NOTE: If reviewer comments were submitted as an attachment file, they will be attached to this email and accessible via the submission site. Please log into your account, locate the manuscript record, and check for the action link "View Attachments". If this link does not appear, there are no attachment files.]

To ensure your figures meet our technical requirements, please review our figure guidelines: https://journals.plos.org/plosone/s/figures

You may also use PLOS’s free figure tool, NAAS, to help you prepare publication quality figures: https://journals.plos.org/plosone/s/figures#loc-tools-for-figure-preparation.

NAAS will assess whether your figures meet our technical requirements by comparing each figure against our figure specifications.

# Author comment: Author response to Decision Letter 1

**DOI:** 10.1371/journal.pone.0335835.r002

8 Jun 2026

Reviewer #1: Review of D-25-56126

Dear Reviewer,

thank you very much for the helpful comments. We changed and responded to them accordingly.

General commentsThe present manuscript presents the results from a Factorial (almost Response Surface) experimental design to study optimal ranges of salinity and light for several strains of picocyanobacterial. The main parameters analyzed as a response to salinity x light were biovolume, chlorophyll a, and fluorescence. The strains were initially selected in order to represent a salinity gradient, based their habitats of origin. In addition, it was performed a genomic study, which main objective was to find patterns of adaptation to the salinity gradient as well.The results show a clear pattern of adaptation in terms of the different measures of growth optima. But there are no main patterns in terms of genomic adaptation.I would recommend the publication of the present manuscript after the correction of several minor aspects detailed below.Specific comments- Line 49: I guess it should be ‘clades’ not ‘claded’.

Changed accordingly, see line 47.- Lines 91-92: This statement, establishing the main hypothesis of this study has to be corrected, since the authors did not perform a strict test for the presence of the meta-populations they referred to. Their findings are restricted to a single cell isolated from each of the habitats. Of course, if the adaptations to salinity found in the strains match the salinity gradient of their habitat of origin (as it was the case for the physiology but not the genomics), that should be explained, most likely, by the presence of adapted meta-populations. But, strictly, the meta-populations were not studied, only one clone from each habitat.In my opinion, this reference to the meta-populations should be made in the discussion, but not set in the hypothesis.

Changed accordingly, see line 98.- Lines 111-112: I don’t understand the reason for growing those two strains in freshwater (when they are originally brackish) and then shifting to 4 ppm salinity.

We changed these sentences accordingly, see line 116. Those two strains were maintained in freshwater at the original culture collection, but we moved them up to higher salinity, as they were growing better in slightly brackish medium.- Line 118: if you used Milli-Q water, you can skip the term “deionized water”.

We would prefer to keep this wording and also not use Milli-Q (as it is a brand name).- Line 130: the authors make a reference here light levels, for the first time. This confusing for the reader, because the light treatment was not previously described, as it was the salinity. It is described in the next sentence. This sentence should go earlier.

Changed accordingly, see line 139.- Line 166: The Gompertz equation does not show in my pdf. Similar problems in lines 186-187, 211-218 and other places throughout the manuscript.

We re-exported it and attached a pdf with a working equation.- Line 369: the “l” of the Cl ion, is written as a superindex.Changed accordingly, see line 403.- Lines 434-451: There is very little, if any, discussion in this paragraph, it is mainly a repetition of things that have been said, differently, in the Results. If it contains new aspects here, they should be anyways moved to the Results.

Thanks for highlighting this issue. We moved parts of the discussion to the results and expanded the discussion with more cited literature. Please refer to the track-changed version to see the differences.

Dear Reviewer,

Thank you very much for those thoughtful and extensive comments. We re-wrote large parts of our results and discussion section and also referred to suggested literature where useful. We respond to specific comments with line numbers. More extensive changes are highlighted in our track-change version.

Reviewer #2: The study by Berthold et al. is a physiological study of salinity and PAR response of five picocyanobacterial strains. The study also investigated the genetic capacity of osmoregulation in the strains. The study is a valuable contribution to the increasing interest in brackish microorganisms diversity and adaptation. I encourage the authors to develop the presentation of their data and the discussion especially in the context of the brackish environment and physiological response of the diverse isolates.In the abstract the authors mention acclimation to different conditions and that Chla can vary. In this context, biovolume may also vary across different conditions. Cell size and temperature relationships are well established in literature, and similarly light and chla, but the physiological response across salinity gradients is less explored to which this study brings new important knowledge. In the current version of the manuscript, the GAM approach, although highly relevant, prevents a discussion on the physiological adaptation in terms of Chl a content, and cell size which I suggest to expand. For example, was there a consistent trend in the adaptation across the different isolates or did it vary depending on metabolic strategies?The introduction and discussion could be improved by including more information about the brackish context in relation to diversity and adaptation to gradients in light, temperature, nutrients and salinity. On the macroorganism level, brackish conditions are associated with smaller organism size (e.g. mussels) and less diversity. However, on the microorganism level, brackish environments harbor a unique and high diversity of organisms. See for example Mazur-Marzec et al. 2024 FEMS Microbiology Reviews, Volume 48, https://doi.org/10.1093/femsre/fuae024; Herlemann et al. 2011 The ISME Journal 5, 571–1579 (2011) https://www.nature.com/articles/ismej201141. Similarly, the brackish picocyanobacterial composition seems to be much more diverse compared to other environments (>2000 16S ASVs Alegria-Zufia et al. 2022 Sci Rep 12:14330. 10.1038/s41598-022-18454-8). Further, in the context of genomic strategies of salinity tolerance the authors may want to refer to the study by Cabello Yeves et al. BMC Biology (2022) 20:175 https://doi.org/10.1186/s12915-022-01379-zLarge parts of the discussion section would be a better fit for the results section. The results section largely describes the patterns observed in the figures but the overall patterns in the results are more clearly presented in the discussion, including a comparison between the strains which I found well written. A more thorough contextualization of the new knowledge presented in this study compared with previous studies and larger implications would also strengthen the discussion. In the abstract, large emphasis is put on metabolic capacity and rate, but this is not well supported in the results and discussion.Furthermore, the section on optical attenuation in the discussion is not well connected with the findings of the study or the knowledge of strain specific light niches and response in pigmentation to salinity and light (see e.g. Aguilera et al. 2023 Environ Microbiol 25: 1674-1695 https://doi.org/10.1111/1462-2920.16384). Here the additional knowledge of the different physiological response among the strains investigated in the study could be more clearly presented.I would find it helpful with a table summarizing the characteristics of each of the tested strains, for example, place of isolation, clade, pigmentation, genome size, original reference, accession number.There was a formatting issue with formulas and symbols being replaced by squares in the version that I have read.Specific comments:Line 43-44: This sentence is written as if a small genome would preclude within clade diversity. Please rephrase.

Changed accordingly, see line 43.Line 47-49: The clade division gets complicated. Rather than mentioning Synechococcus and Cyanobium as a clade I would suggest using the established cluster 5 (5.1, 5.2 and 5.3).

Thanks for the suggestion. We added the information, where available.Line 70: Here you mention salinity but in the context of the paragraph other parameters such as temperature and light may be more relevant.

Changed accordingly, see line 70.Line 81-83: This sentence is unclear. It sounds like there is a problem that there are more isolates and data for these.

Re-phrased the sentence, see line 82.

Line 88-89: All prokaryotes are “related based on conserved sequences including 16s rDNA”. Please be more specific to address why these particular strains were included in the study.

The choice of strains is now addressed:

"..originating from geographically and ecologically diverse regions of the Gulf of Mexico, Baltic Sea, Chesapeake Bay, and coastal Pacific. We chose these particular strains as they are either isolated from similar ecosystems, or are already included in physiological or genomic studies elsewhere, allowing us to validate our findings." see line 91 ff.

Line 91: “across brackish environments” unclear, are you referring to the brackish microbiome or salinity gradients from marine, brackish to freshwater.

Changed accordingly, see line 93.

Line 365: “in study picocyanobacterial” revise to “for the picocyanobacterial in this study”

Changed as suggested., see line 386

Line 372: “different saline environments” unclear.

Changed accordingly., see line 366.

Line 374: Please add a reference.

Added a reference.

Line 383-385: Please see Cabello Yeves et al. BMC Biology (2022) 20:175 https://doi.org/10.1186/s12915-022-01379-z

Thanks, we added that one, too.

Line 419-420: This statement implies that this is the only mechanism. Please rephrase.

Changed while re-writing the section.

Other Changes:

For clarity abbreviations for metabolites glycosyl glycerol, glycosyl glycerate, and glycine betaine were replaced with the full terms.

Attachment Submitted filename: ResponseToReviewers_Berthold_EtAl_PLOSONE.docx

# Peer review: Decision Letter 1

**DOI:** 10.1371/journal.pone.0335835.r003

**Authors:** Balaji Prasath Barathan

8 Jul 2026

Dear Dr. Campbell,

Thank you for submitting your manuscript to PLOS ONE. After careful consideration, we feel that it has merit but does not fully meet PLOS ONE’s publication criteria as it currently stands. Therefore, we invite you to submit a revised version of the manuscript that addresses the points raised during the review process.

Please submit your revised manuscript by Aug 22 2026 11:59PM. If you will need more time than this to complete your revisions, please reply to this message or contact the journal office at plosone@plos.org. When you're ready to submit your revision, log on to https://www.editorialmanager.com/pone/ and select the 'Submissions Needing Revision' folder to locate your manuscript file.

- A letter that responds to each point raised by the academic editor and reviewer(s). You should upload this letter as a separate file labeled 'Response to Reviewers'.
- A marked-up copy of your manuscript that highlights changes made to the original version. You should upload this as a separate file labeled 'Revised Manuscript with Track Changes'.
- An unmarked version of your revised paper without tracked changes. You should upload this as a separate file labeled 'Manuscript'.

If you would like to make changes to your financial disclosure, please include your updated statement in your cover letter. Guidelines for resubmitting your figure files are available below the reviewer comments at the end of this letter.

If applicable, we recommend that you deposit your laboratory protocols in protocols.io to enhance the reproducibility of your results. Protocols.io assigns your protocol its own identifier (DOI) so that it can be cited independently in the future. For instructions see: https://journals.plos.org/plosone/s/submission-guidelines#loc-laboratory-protocols. Additionally, PLOS ONE offers an option for publishing peer-reviewed Lab Protocol articles, which describe protocols hosted on protocols.io. Read more information on sharing protocols at https://plos.org/protocols?utm_medium=editorial-email&utm_source=authorletters&utm_campaign=protocols.

As the corresponding author, your ORCID iD is verified in the submission system and will appear in the published article. PLOS supports the use of ORCID, and we encourage all coauthors to register for an ORCID iD and use it as well. Please encourage your coauthors to verify their ORCID iD within the submission system before final acceptance, as unverified ORCID iDs will not appear in the published article. *Only*  the individual author can complete the verification step; PLOS staff *cannot*  verify ORCID iDs on behalf of authors.

We look forward to receiving your revised manuscript.

Kind regards,

Balaji Prasath Barathan

Academic Editor

PLOS One

Journal Requirements:

If the reviewer comments include a recommendation to cite specific previously published works, please review and evaluate these publications to determine whether they are relevant and should be cited. There is no requirement to cite these works unless the editor has indicated otherwise.

Please review your reference list to ensure that it is complete and correct. If you have cited papers that have been retracted, please include the rationale for doing so in the manuscript text, or remove these references and replace them with relevant current references. Any changes to the reference list should be mentioned in the rebuttal letter that accompanies your revised manuscript. If you need to cite a retracted article, indicate the article’s retracted status in the References list and also include a citation and full reference for the retraction notice.

[Note: HTML markup is below. Please do not edit.]

Reviewers' comments:

Reviewer's Responses to Questions

**Comments to the Author**

Reviewer #1: All comments have been addressed

Reviewer #2: (No Response)

**********

2. Is the manuscript technically sound, and do the data support the conclusions?

Reviewer #1: Yes

Reviewer #2: Yes

**********

3. Has the statistical analysis been performed appropriately and rigorously?

Reviewer #1: Yes

Reviewer #2: Yes

**********

4. Have the authors made all data underlying the findings in their manuscript fully available?

The PLOS Data policy

Reviewer #1: Yes

Reviewer #2: Yes

**********

5. Is the manuscript presented in an intelligible fashion and written in standard English?

Reviewer #1: Yes

Reviewer #2: Yes

**********

Reviewer #1: All the comments, which were not many, and critical, were fully addressed. In particular, being the most important the one about removing the term "Meta-populations" from the hypotheses.

Reviewer #2: Thank you for the revision. I have previously reviewed this manuscript and I believe that it is a valuable contribution with solid experimental data addressing coupling between physiological adaptation and genomics of brackish picocyanobacteria.

Although the authors have responded to my minor specific comments, they have not clearly addressed my general comments in the response letter or in the revised manuscript. This includes a suggestion of expanding the discussion on the physiological adaptation in terms of Chl a content, and cell size as well as a more thorough description about the brackish context in relation to diversity and adaptation to gradients in light, temperature, nutrients and salinity. I understand that not all suggestions have to be fully integrated in a revised manuscript but I would appreciate a clear response from the authors.

I also encourage the authors to include a conclusion about their findings and the larger implications at the end of the discussion. The last paragraph of the discussion is now on the optical attenuation, which was a section that I encouraged the authors to expand on, but it may not be the final message that the authors want to convey (does not align with the abstract or aims in the introduction).

There is still a problem with formulas and special signs as they show as boxes in my version (which was also pointed out by Reviewer#1).

**********

what does this mean?). If published, this will include your full peer review and any attached files.

If you choose “no”, your identity will remain anonymous but your review may still be made public.

**Do you want your identity to be public for this peer review?**  For information about this choice, including consent withdrawal, please see our Privacy Policy

Reviewer #1: No

Reviewer #2: No

**********

[NOTE: If reviewer comments were submitted as an attachment file, they will be attached to this email and accessible via the submission site. Please log into your account, locate the manuscript record, and check for the action link "View Attachments". If this link does not appear, there are no attachment files.]

To ensure your figures meet our technical requirements, please review our figure guidelines: https://journals.plos.org/plosone/s/figures

You may also use PLOS’s free figure tool, NAAS, to help you prepare publication quality figures: https://journals.plos.org/plosone/s/figures#loc-tools-for-figure-preparation.

NAAS will assess whether your figures meet our technical requirements by comparing each figure against our figure specifications.

# Author comment: Author response to Decision Letter 2

**DOI:** 10.1371/journal.pone.0335835.r004

21 Aug 2026

Reviewer 2

Although the authors have responded to my minor specific comments, they have not clearly addressed my general comments in the response letter or in the revised manuscript. This includes a suggestion of expanding the discussion on the physiological adaptation in terms of Chl a content, and cell size as well as a more thorough description about the brackish context in relation to diversity and adaptation to gradients in light, temperature, nutrients and salinity. I understand that not all suggestions have to be fully integrated in a revised manuscript but I would appreciate a clear response from the authors.

We expanded the discussion now to include cell size influences potential colony formation. However, we did not expand the discussion on Chl a content, temperature or nutrients besides what is already present in the manuscript. We see them as important, but they were not the major focus of this study. After this salinity-focussed data set we accumulated similar datasets on temperature, nutrients and photoperiods for manuscript(s) in the future, but would be too much for just one manuscript.

From your first review: In the current version of the manuscript, the GAM approach, although highly relevant, prevents a discussion on the physiological adaptation in terms of Chl a content, and cell size which I suggest to expand. For example, was there a consistent trend in the adaptation across the different isolates or did it vary depending on metabolic strategies?

We added individual GAMs for each strain to the supplement, to make strain-specific trends easier to see. Also, we highlight, that Supplement Table 2 includes strain specific biovolume estimates for each light level.

I also encourage the authors to include a conclusion about their findings and the larger implications at the end of the discussion. The last paragraph of the discussion is now on the optical attenuation, which was a section that I encouraged the authors to expand on, but it may not be the final message that the authors want to convey (does not align with the abstract or aims in the introduction).

Thank you again for the opportunity. We agree and have added a small Conclusions section to the manuscript.There is still a problem with formulas and special signs as they show as boxes in my version (which was also pointed out by Reviewer#1).

We checked again with our own generated .pdfs, where the formulas are visible. The issue arises when the PLoS Manuscript submission system auto-generates a .pdf file from our .docx. We have uploaded the manuscript as pdf with this submission and will talk with the editorial board about the issue if the manuscript goes into production.

Attachment Submitted filename: ResponseToReviewers_Berthold_EtAl_PLOSONE_Rev2.docx

# Editor report: Decision Letter 2

**DOI:** 10.1371/journal.pone.0335835.r005

**Authors:** Balaji Prasath Barathan

16 Sep 2026

Inter-strain diversity among coastal picocyanobacteria across salinity gradients

PONE-D-25-56126R2

Dear Dr. Douglas,

We’re pleased to inform you that your manuscript has been judged scientifically suitable for publication and will be formally accepted for publication once it meets all outstanding technical requirements.

Within one week, you’ll receive an e-mail detailing the required amendments. When these have been addressed, you’ll receive a formal acceptance letter and your manuscript will be scheduled for publication.

An invoice will be generated when your article is formally accepted. Please note, if your institution has a publishing partnership with PLOS and your article meets the relevant criteria, all or part of your publication costs will be covered. Please make sure your user information is up-to-date by logging into Editorial Manager at Editorial Manager® and clicking the ‘Update My Information' link at the top of the page. For questions related to billing, please contact billing support.

If your institution or institutions have a press office, please notify them about your upcoming paper to help maximize its impact. If they’ll be preparing press materials, please inform our press team as soon as possible -- no later than 48 hours after receiving the formal acceptance. Your manuscript will remain under strict press embargo until 2 pm Eastern Time on the date of publication. For more information, please contact onepress@plos.org.

Kind regards,

Balaji Prasath Barathan

Academic Editor

PLOS One

Additional Editor Comments (optional):

Reviewers' comments:

# Editor report: Acceptance letter

**DOI:** 10.1371/journal.pone.0335835.r006

**Authors:** Balaji Prasath Barathan

PONE-D-25-56126R2

PLOS One

Dear Dr. Campbell,

I'm pleased to inform you that your manuscript has been deemed suitable for publication in PLOS One. Congratulations! Your manuscript is now being handed over to our production team.

At this stage, our production department will prepare your paper for publication. This includes ensuring the following:

* All references, tables, and figures are properly cited

* All relevant supporting information is included in the manuscript submission,

* There are no issues that prevent the paper from being properly typeset

You will receive further instructions from the production team, including instructions on how to review your proof when it is ready. Please keep in mind that we are working through a large volume of accepted articles, so please give us a few days to review your paper and let you know the next and final steps.

Lastly, if your institution or institutions have a press office, please let them know about your upcoming paper now to help maximize its impact. If they'll be preparing press materials, please inform our press team within the next 48 hours. Your manuscript will remain under strict press embargo until 2 pm Eastern Time on the date of publication. For more information, please contact onepress@plos.org.

You will receive an invoice from PLOS for your publication fee after your manuscript has reached the completed accept phase. If you receive an email requesting payment before acceptance or for any other service, this may be a phishing scheme. Learn how to identify phishing emails and protect your accounts at https://explore.plos.org/phishing.

If we can help with anything else, please email us at customercare@plos.org.

Thank you for submitting your work to PLOS One and supporting open access.

Kind regards,

PLOS One Editorial Office Staff

on behalf of

Dr. Balaji Prasath Barathan

Academic Editor

PLOS One
